# Every Gain Comes With Loss: Ecological and Physiological Shifts Associated With Polyploidization in a Pygmy Frog

**DOI:** 10.1093/molbev/msaf037

**Published:** 2025-02-07

**Authors:** Qiheng Chen, Wei Zhu, Liming Chang, Meihua Zhang, Shouhong Wang, Jiongyu Liu, Ningning Lu, Cheng Li, Feng Xie, Bin Wang, Jianping Jiang

**Affiliations:** Chengdu Institute of Biology, Chinese Academy of Sciences, Chengdu, China; Chengdu Institute of Biology, Chinese Academy of Sciences, Chengdu, China; Chengdu Institute of Biology, Chinese Academy of Sciences, Chengdu, China; Chengdu Institute of Biology, Chinese Academy of Sciences, Chengdu, China; Chengdu Institute of Biology, Chinese Academy of Sciences, Chengdu, China; Chengdu Institute of Biology, Chinese Academy of Sciences, Chengdu, China; Chengdu Institute of Biology, Chinese Academy of Sciences, Chengdu, China; Chengdu Institute of Biology, Chinese Academy of Sciences, Chengdu, China; Chengdu Institute of Biology, Chinese Academy of Sciences, Chengdu, China; Chengdu Institute of Biology, Chinese Academy of Sciences, Chengdu, China; Chengdu Institute of Biology, Chinese Academy of Sciences, Chengdu, China

**Keywords:** adaptation, autotetraploid, ecological differentiation, speciation, whole-genome duplication

## Abstract

Polyploidization plays a pivotal role in vertebrate evolution and diversification. However, the effects of polyploidization on animals across various biological levels, and how these differences drive ecological shifts, remain unclear. Through karyotype analysis and whole-genome sequencing, we identified an autotetraploid *Microhyla fissipes* from Hainan Island, which shows reproductive isolation and geographic differentiation from its diploid counterpart. Tetraploids exhibited larger cell size, improved tadpole growth rates, and greater whole-body size, along with reduced cell cycle activity. Rather than being simple scaled-up diploids, tetraploids showed shifts in physiological performance, organ allometry, gene expression profiles, and metabolic patterns. Tetraploid adults demonstrated superior jumping ability and increased reproductive investment (e.g. larger gonads and steeper slopes in the relationship between gonadal weight and body weight), suggesting a potential competitive advantage over diploids. However, tetraploids exhibited higher energy expenditure at elevated temperatures, reduced hepatic energy storage, and altered pulmonary regulatory metabolites at 25 °C. Males had smaller relative heart sizes, and females showed flatter slopes in the relationship between heart and lung weight and body weight, indicating reduced investment in cardiopulmonary system. These variations suggest an increased risk of metabolic constraints under heat stress, putting tetraploids at a disadvantage in warmer regions. Importantly, the physiological tradeoffs associated with polyploidization help explain the geographical differentiation between diploids and tetraploids, which reflects a climatic boundary, with tetraploids occupying cooler northeastern areas. Our findings identify an autotetraploid frog, report the first autotetraploid genome in amphibians, and demonstrate how vertebrate polyploids physiologically and ecologically diverge from their diploid counterparts.

## Graphical Abstract

**graphical abstract:**
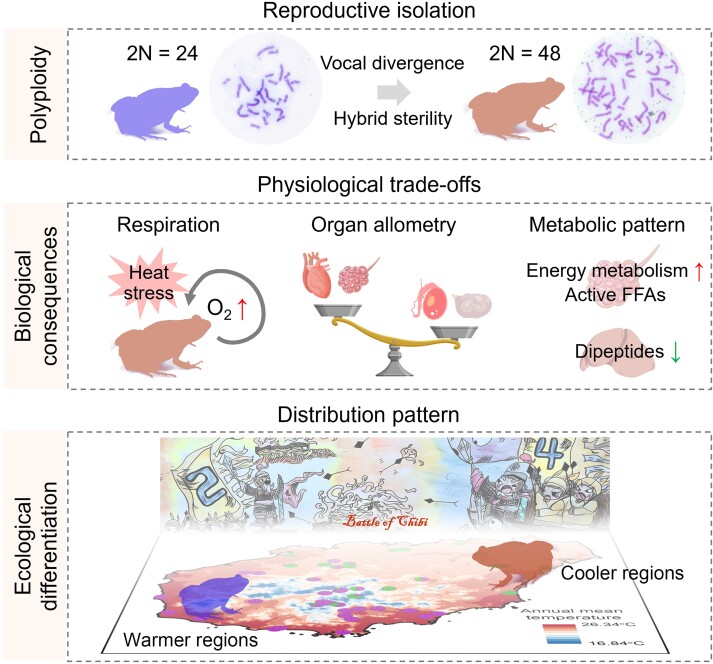

## Introduction

Polyploidy, or whole-genome duplication (WGD), is a key mechanism in species formation and has been shown to be crucial to vertebrate evolution (Angel et al. 1998; Marlétaz et al. 2024). Two ancient WGD events lay the groundwork for the current diversity of vertebrate (Dehal and Boore 2005; Yu et al. 2024). Among extant vertebrates, current polyploidy rarely occurs in birds and mammals, and relatively more common in fishes (Li and Guo 2020) and amphibians (Schmid et al. 2015). These polyploidy phenomena offer unique models for understanding the mechanisms driving vertebrate evolution and the formation of biodiversity.

The fate of polyploidy, along with its advantages and disadvantages compared to the diploid counterpart, remains controversial. The rarity of polyploid species in long-term evolution makes polyploidy to be considered as an evolutionary “dead end” in most cases (Wagner 1970; Arrigo and Barker 2012). This is because polyploidy likely brings detrimental effects on fertility and fitness due to genomic instability, mitotic and meiotic abnormalities (Comai 2005). Nevertheless, polyploidy is believed to provide better adaptability to harsh environments than their closely related diploids (te Beest et al. 2012; Van de Peer et al. 2017; Li and Guo 2020). Duplicated genes can undergo subfunctionalization and neofunctionalization (Lynch and Conery 2000; Adams and Wendel 2005), fueling long-term diversification and preventing large drops in fitness during environmental upheavals (Van de Peer et al. 2009; Li et al. 2024). This is supported by the fact that WGDs appear to correlate with periods of extinction or global change, and the establishment and success of polyploid lineages often occur in harsh or disturbed environments (Mable et al. 2011; Ren et al. 2018; Van de Peer et al. 2021). Theoretically, the success or failure of polyploidy may not be predetermined but depends on how well the biological outcomes of genome duplications align with historical and current environmental conditions. Achieving a comprehensive understanding of polyploidy requires not only a thorough investigation of its biological consequences but also an exploration of the ecological effects of these variations.

Physiological changes provide critical insights into shifts in species' adaptability to environmental conditions, potentially driving ecological differentiation. Variations in body size and metabolic rate are 2 widely studied physiological effects of WGDs. Though polyploidization consistently results in enlarged cells (Olmo 1983; Futcher and Kellogg 2024), its effect on animal's overall body size varies considerably. In nematodes, polyploidy is reported to result in increased body size (Triantaphyllou and Hirschmann 1997; Flemming et al. 2000). In vertebrates such as mice (Henery et al. 1992), frogs (Cadart et al. 2023), and salamanders (Fankhauser 1945), body size remains unchanged after polyploidization due to a compensatory reduction in cell number. The effect of polyploidy on whole-body metabolic rate (i.e. oxygen consumption) in vertebrates is independent on changes in body size (Atkins and Benfey 2008; Polymeropoulos et al. 2014; Hermaniuk et al. 2017, 2021; Cadart et al. 2023). Polyploid *Xenopus* have lower mass-specific metabolic rate due to reduced energy requirements for maintaining cell membranes and ionic gradients, resulting from a lower cell surface area-to-volume ratio (Cadart et al. 2023). Nevertheless, it remains inconclusive whether polyploid vertebrates consistently exhibit a lower metabolic rate (Hermaniuk et al. 2021). In addition to whole-body parameters, the variation in relative organ size and allometric growth patterns are also indicative to potential ecological differentiation in animals' life strategy (e.g. resource allocation between reproduction and storage) (Griffen et al. 2018; Zhong et al. 2018; Huang et al. 2020), but remains under-studied.

Physiological changes associated with polyploidy are inherently dependent on variations in gene expression patterns, which have been well documented in plants (te Beest et al. 2012; Soltis et al. 2014; McCarthy et al. 2016). These biological variations can result from intrinsic alterations in the central dogma due to increased genetic material and enlarged nuclei, or from the reorganization of genomic architecture after long-term evolution (Comai 2005). For instance, in the allotetraploid frog *Xenopus laevis*, the S chromosomes are shorter than their L counterparts in karyotypic measurements (13.2%) and assembled sequences (17.3%) (Matsuda et al. 2015; Session et al. 2016). Additionally, genes from the S and L subgenomes exhibit different expression patterns during development (Session et al. 2016). Similar cases have been reported in allopolyploid carp (*Cyprinus carpio*) (Xu et al. 2019). Revealing the transcriptional changes induced by polyploidization can provide deeper insights into its physiological consequences and the mechanisms underlying their formation.

Despite significant progress in understanding the biological effects of polyploidy and its underlying mechanisms, whether and how these changes are connected to potential ecological consequences remains largely unexplored. This includes their impact on competition between polyploids and diploids, as well as their influence on environmental adaptability and niche differentiation. Indeed, it is impossible to determine whether biological changes are advantageous or detrimental without considering the environmental context. The ecological implications of such changes become meaningful only when examined within the framework of specific environmental factors, such as microhabitats and climate. Therefore, an ideal model for addressing these knowledge gaps should ensure that both tetraploids and their closely related diploids persist within their natural distribution ranges, potentially engaging in competitive interactions or exhibiting differences in geographic differentiation.

Here, we report the discovery of a recently occurred autotetraploid amphibian, *Microhyla fissipes*, which underwent polyploidization ∼4.04 million years ago on Hainan Island. These tetraploids exhibit significant morphological and geographic differences compared to their closely related diploids. Using a combination of whole-genome sequencing, comparative multi-omics, and climatic association analyses, we investigated the biological outcomes of polyploidy in *M. fissipes*, as well as the tradeoffs of polyploidy in competition with diploids in the context of environmental adaptation. We hypothesize that: (i) polyploidy induces changes across multiple biological levels (e.g. physiology, transcription, and metabolism), influencing the survival strategies and environmental adaptability; (ii) the biological consequences of polyploidy are not inherently advantageous or detrimental, but involve tradeoffs shaped by environmental conditions, driving ecological differentiation between diploids and tetraploids. We hope this study provides a comprehensive example of how polyploidy can drive ecological differentiation and shape interactions between polyploids and their closely related diploids.

## Results

### Identification of Autotetraploid *M. fissipes*

**Fig. 1.**
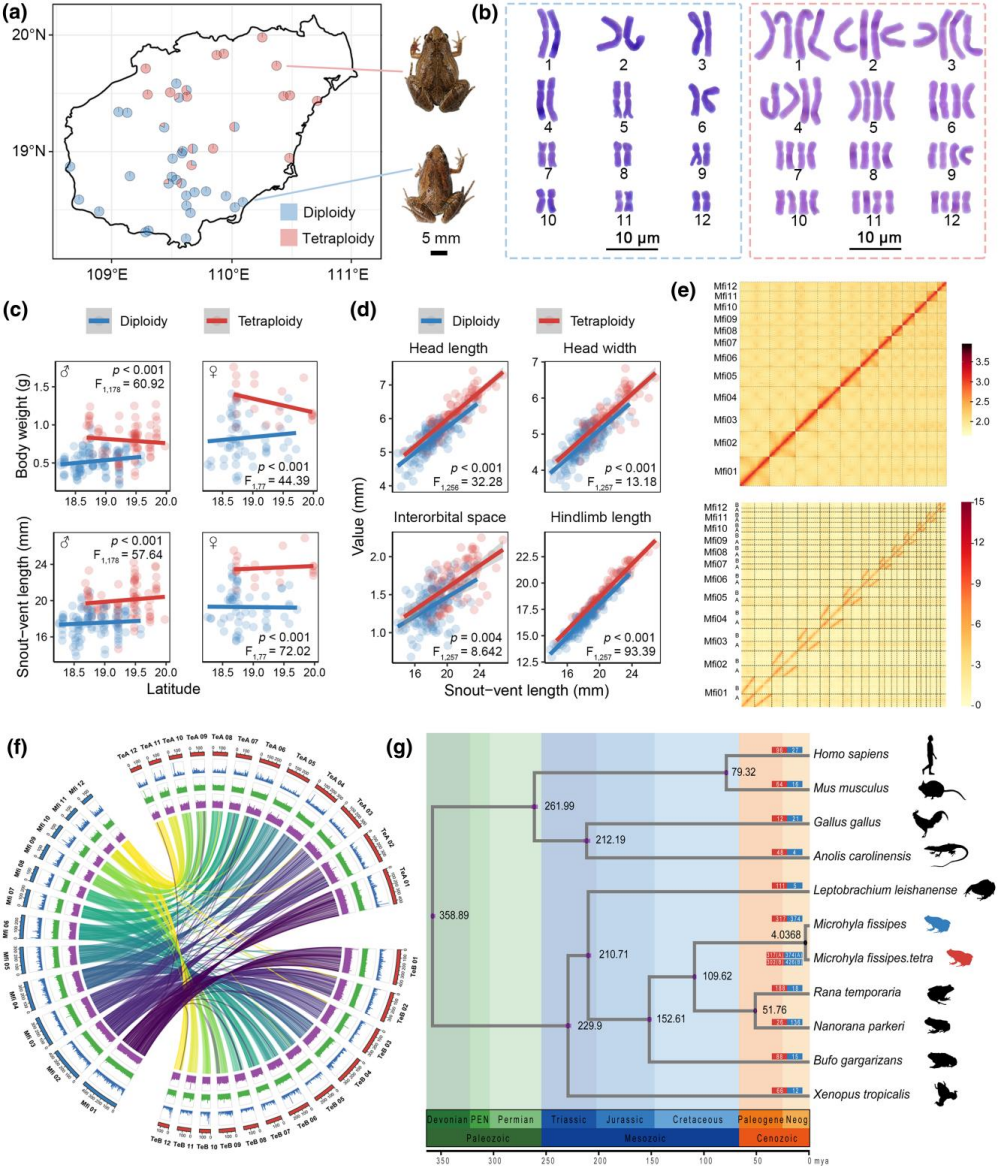
Identification of tetraploid *M. fissipes*. a) Collection sites of the diploid and tetraploid *M. fissipes*. b) Typical karyotype of the diploid and tetraploid *M. fissipes*. c) Comparison of body weight and SVL between diploid and tetraploid individuals. The data were analyzed with ANOCOVA, with ploidy and latitude considered as fixed factor and covariate, respectively. d) Relative body size of the diploid and tetraploid individuals. The data were analyzed with ANOCOVA, with ploidy and sex considered as fixed factor, and SVL as covariate. e) Hi-C interactions among chromosomes of diploid and tetraploid, respectively. f) Chromosome synteny between diploid and tetraploid. g) Phylogenetic analysis and estimation of divergence time.

*Microhyla fissipes* typically have 12 pairs of chromosomes (2n = 24) in their somatic cells. However, a comprehensive survey across 54 locations (supplementary table S1, Supplementary Material online) on Hainan Island revealed two karyotypes in these animals, which exhibit no obvious morphological differences: one with the traditional 12 pairs and the other with 24 pairs (both provisionally referred to as *M. fissipes* in this study; Fig. 1a and b and supplementary fig. S1, Supplementary Material online). Both karyotypes share the same set of 12 haplotype groups, and chromosomes within each group exhibited striking morphological similarity (Fig. 1b and supplementary table S2, Supplementary Material online). Accordingly, the individuals were identified as diploid (12 pairs) and autotetraploid (24 pairs). Tetraploids were significantly larger in overall body size, with relatively larger heads and longer hindlimbs than the diploids (Fig. 1c and d). The two karyotypes exhibit distinct geographical distributions in Hainan Island, with diploids predominantly in the southeast and tetraploids in the northwest, though overlapping regions exist (Fig. 1a).

We assembled chromosome-level genome for both diploid and tetraploid *M. fissipes* (supplementary tables S3 and S4, Supplementary Material online), yielding 12 scaffolds (3.31 GB) for diploids and 24 scaffolds (6.00 GB) for tetraploids (Fig. 1e and supplementary fig. S2, Supplementary Material online). The two subsets of pseudochromosomes in tetraploids exhibited high structural similarity, with extensive synteny and similar chromosomal landscapes to diploid pseudochromosomes (Fig. 1f). This indicates that the tetraploid subgenomes likely originated from the same diploid ancestor. Comparative genomic analyses identified 21,515 shared gene families and 1,235 single-copy orthologous genes across *M. fissipes*, five other anurans (*Rana temporaria*, *Nanorana parkeri*, *Bufo gargarizans*, *Leptobrachium leishanense*, and *Xenopus tropicalis*), and four nonamphibian vertebrates (*Mus musculus*, *Homo sapiens*, *Gallus gallus*, and *Anolis carolinensis*). Divergent analysis based on single-copy orthologs estimated that diploid and tetraploid *M. fissipes* split ∼4.04 million years ago (Fig. 1g and supplementary fig. S3, Supplementary Material online), while the tetraploid subgenomes diverged ∼3.59 million years ago (supplementary fig. S3, Supplementary Material online). This supports the origin of tetraploids from a single ancestral diploid genome rather than hybridization between different species.

**Fig. 2.**
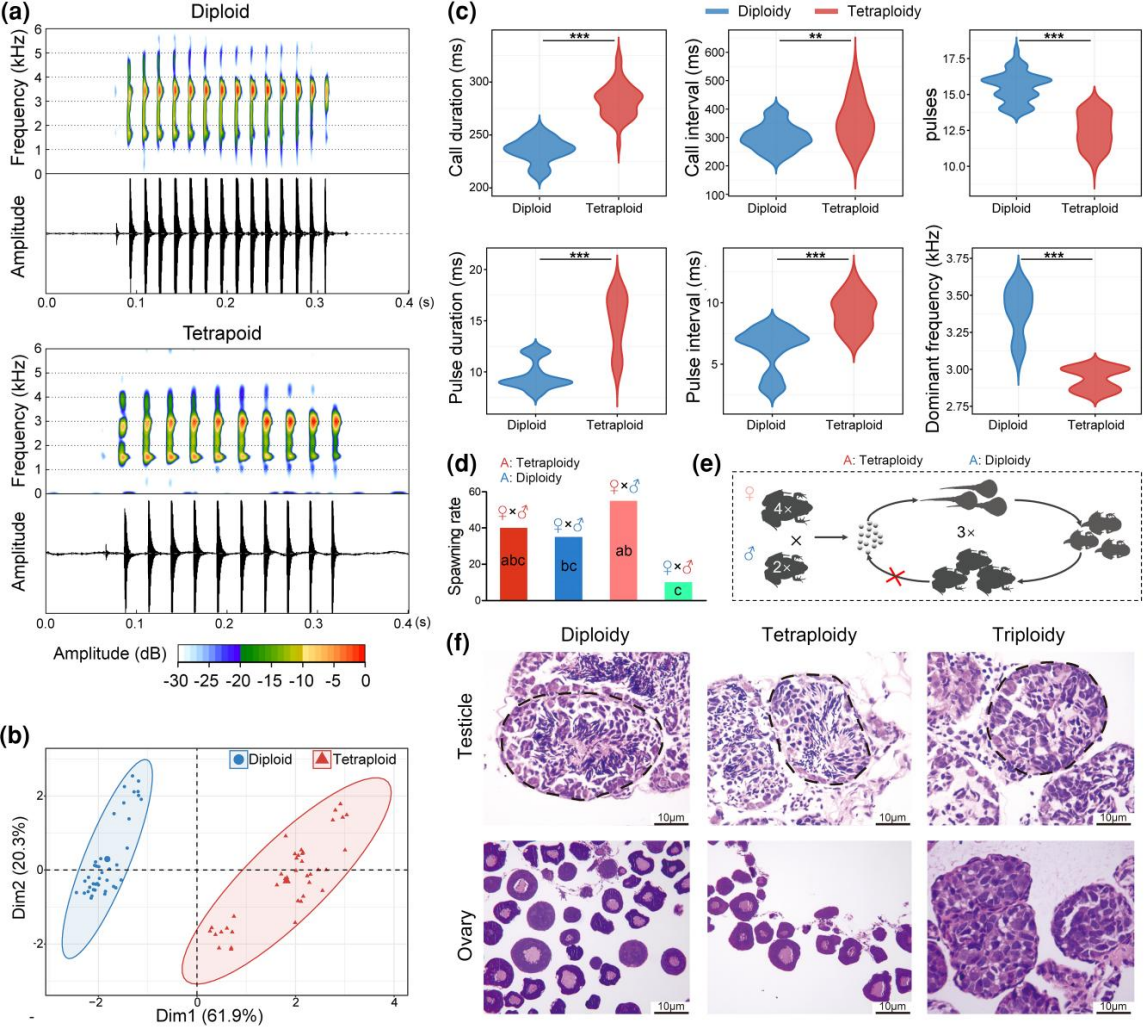
Reproductive isolation between diploid and tetraploid *M. fissipes*. a) Typical advertisement calls of diploid and tetraploid *M. fissipes*. b) PCA displaying the divergence in the characteristics of advertisement calls between diploid and tetraploid *M. fissipes*. Each point denotes a call, and 15 calls were recorded for each individual (eight individuals per group). c) Differences in the characteristics of advertisement calls between diploid and tetraploid *M. fissipes*. **, *P* < 0.01; ***, *P* < 0.001 (Wilcoxon-test). d) Spawning rate of different parental combinations. The data were analyzed with χ^2^ test followed by Z test. Different letters denote significant differences between groups. e) Schematic diagram illustrating the reproductive isolation between diploid males and tetraploid females. The offspring produced by a tetraploid mother and a diploid father can grow normally, but their gonads do not develop properly, leading to reproductive isolation between the tetraploid and diploid parents. f) Histological characteristics of testicle and ovary. The seminiferous tubules were highlighted with dashed line.

We did not identify any triploid individuals after examining the karyotype of 261 wild specimens, including 105 collected from regions where diploid and tetraploid distributions overlap. Male advertisement calls differed significantly between diploids and tetraploids (Fig. 2a to c and supplementary fig. S4, Supplementary Material online), a key speciation indicator. In controlled matings, spawning rates remained stable for diploid males and tetraploid females but dropped significantly when pairing tetraploid males with diploid females. Although F1 triploids reached adulthood (supplementary fig. S5, Supplementary Material online), their ovaries and testes failed to develop normally (Fig. 2f and supplementary fig. S6 and S7, Supplementary Material online). This indicates reproductive isolation between diploids and tetraploids.

### Divergence in Tadpole Growth and Metabolism

**Fig. 3.**
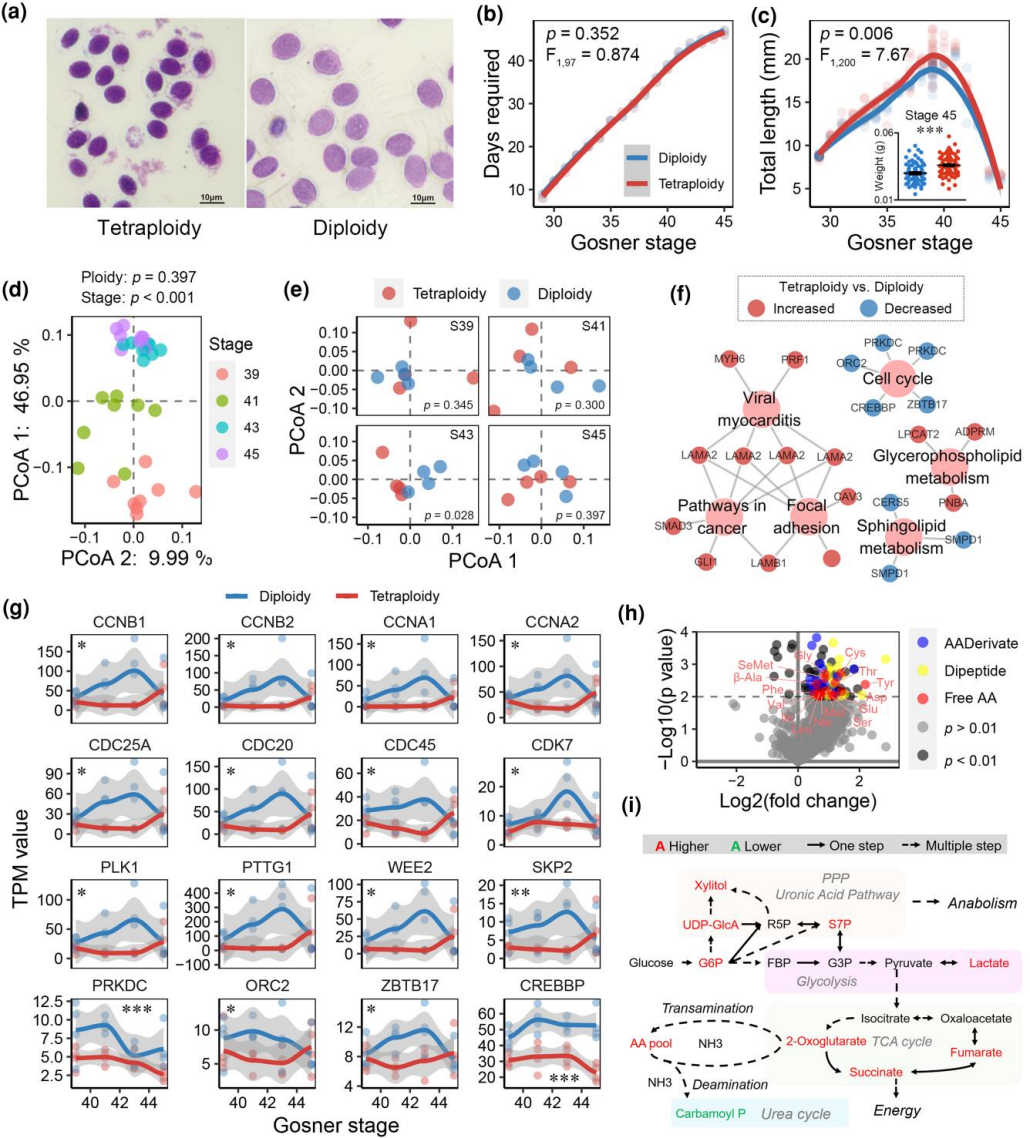
Molecular insight into the different growth rates of diploid and tetraploid tadpoles. a) Comparison of the size of red blood cells. b and c) Comparison of the development (b) and growth (c) rates between diploid and tetraploid tadpoles. The data were analyzed using linear mixed model, with ploidy as a fixed factor, Gosner stage as a covariate, and replicate as a random factor. The text denotes the effects of ploidy. *** in subpanel indicates *P* < 0.001 in Student's *t*-test. d) PCoA on the tadpole transcriptomes. The data were analyzed with PERMANOVA. Developmental stage was a predominant factor in shaping the gene transcription. e) PCoA on tadpole transcriptomes at each developmental stage. The data were analyzed with PERMANOVA. f) Network illustrating the DEGs and enriched KEGG pathways between diploid and tetraploid tadpoles at the same stage. The threshold for DEGs was set at *P* < 0.01 (Students' *t*-test), and for enriched pathways, the adjusted *P* < 0.01 (KOBAS 3.0). Large nodes represent KEGG items, while small nodes represent DEGs. Red and blue DEGs indicate higher transcriptional levels in tetraploid and diploid individuals, respectively. g) Transcriptional pattern of genes involved in cell cycle. The data were analyzed with ANOCOVA: *, *P* < 0.05; **, *P* < 0.01; ***, *P* < 0.001. h) Volcano plot presenting the differential metabolites between diploid and tetraploid tadpoles at Gosner stage 39. i) Difference (tetraploid vs. diploid, *P* < 0.05) in metabolic patterns between diploid and tetraploid tadpoles at Gosner stage 39.

Tetraploid *M. fissipes* showed increased cell and body sizes (Figs. 1c and 3a, and supplementary fig. S8, Supplementary Material online). To explore the mechanisms behind their larger body size, we compared postembryonic development (metamorphosis in amphibians) between tetraploids and diploids. Their metamorphosis rates were synchronized (Fig. 3b), but tetraploids were consistently larger, particularly at stage 39, when the tadpoles reached maximum size, establishing a size advantage in froglets (stage 45) (Fig. 3c).

We conducted a whole-tissue transcriptomic comparison of diploids and tetraploids at key developmental stages (stage 39, 41, 43, and 45) (Fig. 3d), with most significant transcriptional difference observed at stage 43, the metamorphic climax (Fig. 3e). Enrichment analyses of differently expressed genes (DEGs) revealed a transcriptional downregulation of cell cycle (e.g. *CREBBP*, *PRKDC*, and *CDCs*) in the tetraploids across developmental stages (Fig. 3f and g), indicating reduced cell proliferation activity during metamorphosis.

We further compared the whole-tissue metabolome of diploid and tetraploid tadpoles at stage 39. Tetraploids showed higher relative abundance of amino acids, dipeptides, and fatty acids (Fig. 3h), indicating better nutrient status (Zhu et al. 2019). This was coupled with a decrease in the relative abundance of carbamoyl phosphate, a critical metabolite in urea cycle (Matsumoto et al. 2019), suggesting reduced deamination and potentially explaining the elevated amino acid levels in tetraploids. Additionally, tetraploids exhibited increased intermediates in the pentose phosphate pathway (e.g. sedoheptulose 7-phosphate) and uronic acid pathway (e.g. UDP-glucuronic acid) (Fig. 3i), both crucial for biosynthesis, and in the TCA cycle (e.g. 2-oxoglutarate, succinate, and fumarate), an obligate aerobic energy pathway. These results suggest divergent metabolic patterns between diploid and tetraploid tadpoles.

### Divergence in Adult Physiology and Metabolism

**Fig. 4.**
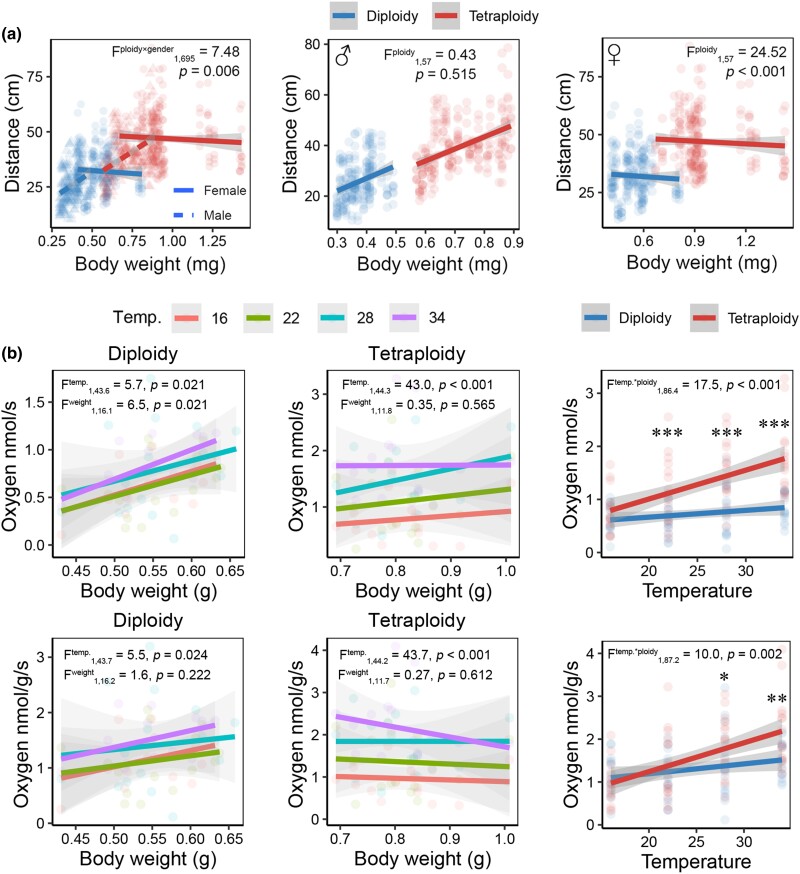
Comparison of physiological traits between diploid and tetraploid *M. fissipes*. a) Jumping performance. The data were analyzed using mixed linear model, with ploidy and gender (left panel) as fixed factors, individuals as random factor, and body weight as covariate. The insignificant interactive effects were removed from the final models. The text describes the effects of ploidy if the interaction was insignificant, or it presents the effects of interaction. b) Respiration rate. When diploidy and tetraploidy were considered separately, the data were analyzed using mixed linear model, with temperature as a fixed factor, body weight as a covariate, and individual as a random factor. When diploidy and tetraploidy were analyzed in the same model, we considered the temperature and ploidy as fixed factors, and individual as a random factor. Simple effect analyses were conducted to compare the intergroup difference at each temperature (*, *P* < 0.05; **, *P* < 0.01; ***, *P* < 0.001), if the interactions between temperature and ploidy were significant.

We compared the physiological performance of tetraploid and diploid *M. fissipes* across several metrics. Jumping distance was influenced by both ploidy and gender (Fig. 4a). In males, jumping distances increased with body size. Tetraploids showed superior absolute jumping performance, though their relative jumping performance (scaled to body size) was comparable to diploids. In females, jumping distance was constant and independent of body size, with tetraploids consistently outperforming diploids. We measured the basic metabolic rate (oxygen consumption rate) of males across a temperature gradient (16, 22, 28, and 34 °C) (Fig. 4b). Without body mass adjustment, metabolic rate increased with body weight only in diploids. After adjusting for body weight (oxygen consumption rate per unit body mass), metabolic rate became independent of body mass in both groups. Both diploids and tetraploids showed increased metabolic rate with temperature (*P* < 0.05), but the increase was more pronounced in tetraploids (interaction: *P* < 0.05), resulting in significantly higher metabolic rates at 28 °C and 34 °C (Fig. 4b). These findings suggest tetraploids have a higher energy budget for maintaining homeostasis under higher temperatures.

**Fig. 5.**
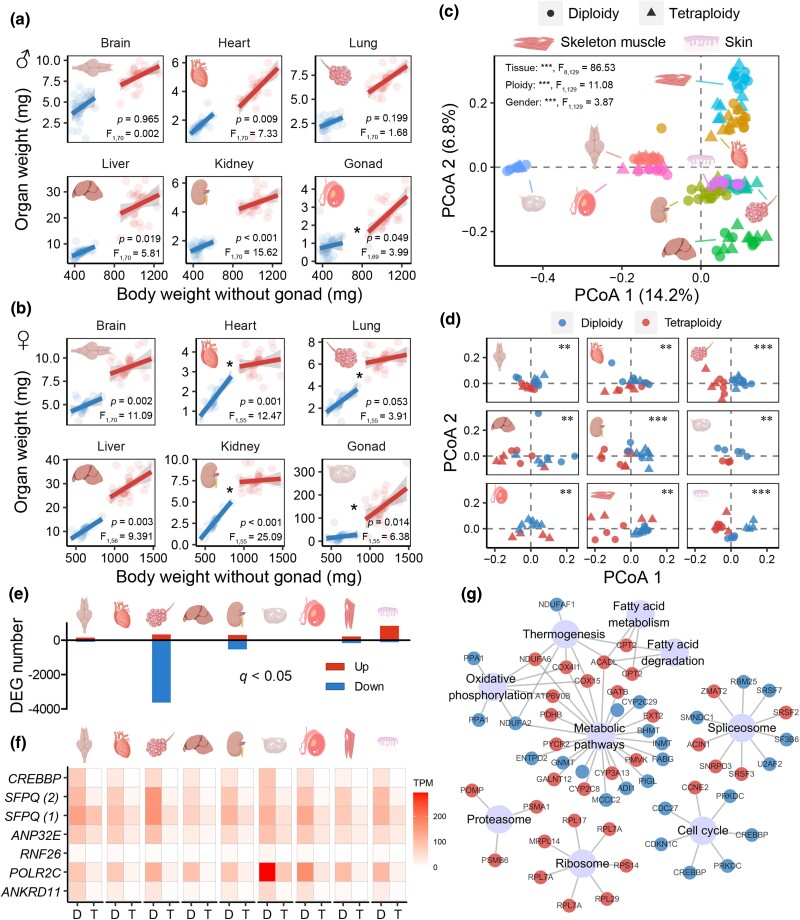
Organ-level differences between diploid and tetraploid adults. a and b) The relationship between organ and whole-body weights in males (a) and females (b). The data were analyzed using ANCOVA (fixed factor: ploidy; covariate: body weight). The body weight values exclude gonad weight due to the gonads' significant and highly variable mass. The insignificant interactive effects were removed from the final models. Asterisks indicate significant interactions between ploidy and body weight. The text describes the effects of ploidy if the interaction was insignificant, or it presents the interaction between ploidy and body weight. c) PCoA scatter plot presenting the dissimilarity in the transcriptome of different organs. The data were analyzed using PERMANOVA, with tissue, ploidy, and gender as independent factors. d) PCoA scatter plot presenting the transcriptional dissimilarity between diploid and tetraploid samples for each organ. The data were analyzed using PERMANOVA, with ploidy and gender as independent factors. e) The numbers of DEGs of each tissue at threshold of adjusted *P* < 0.05 (Student's *t-*test and BH correction). f) DEGs shared by all the nine organs (*P* < 0.01, Student's *t-*test). g) Network presenting the KEGG pathways (adjusted *P* < 0.01) enriched by DEGs (*P* < 0.05, Student's *t-*test) shared by at least six organs. Large nodes represent KEGG items, while small nodes represent DEGs. Red and blue DEGs indicate higher transcriptional levels in tetraploid and diploid individuals, respectively.

Analyses of organ-to-body weight relationship (excluding the gonads due to their considerable individual variations) for diploids and tetraploids revealed that tetraploids are not simply scaled-up versions of diploids (ANCOVA; Fig. 5a and b). In males, after scaling to body weight, tetraploids had larger livers and kidneys but smaller hearts than diploids (*P*^interaction^ > 0.05 and *P*^ploidy^ < 0.05). A significant difference was found in the testicle-to-body weight slopes, with tetraploids showing a greater increase in testicle weight relative to body weight than diploids (*P*^interaction^ < 0.05). In females, tetraploids had larger brains and livers than diploids after scaling to body weight (*P*^interactio*n*^ > 0.05 and *P*^ploidy^ < 0.05). The organ-to-body weight slopes for the ovary, heart, lungs, and kidneys differed between diploids and tetraploids (*P*^interaction^ < 0.05). Tetraploids showed a more pronounced increase in ovary weight, whereas the increase in the heart, lungs, and kidneys relative to body weight was less marked (Fig. 5b). These differences indicate divergent resource allocation strategies between tetraploid and diploid females.

Transcriptomic analysis of nine organs (i.e. brain, heart, lung, liver, kidney, ovary, testicle, muscle, and skin) revealed significant ploidy-dependent alterations in organ transcriptional profiles (Fig. 5c and d). Gender was excluded from subsequent differential analyses (tetraploids vs. diploids) due to its relatively minimal impact on organ transcriptomes. The lung exhibited the greatest transcriptional differences, with most DEGs downregulated in tetraploids (up: down = 328: 3,634; adjusted *P* < 0.05), followed by the skin (up: down = 827: 124) and kidney (up: down = 296: 536) (Fig. 4e). The seven shared DEGs (i.e. *CREBBP*, *SPFQ*, *ANP32E*, *RNF26*, *PLOR2C*, and *ANKRD11*; *P* < 0.01) across all nine organs were downregulation in tetraploids (Fig. 4f), with most of these genes involved in the positive regulation of the cell cycle. Downregulated DEGs (*P* < 0.05) shared by at least six organs revealed transcriptional downregulation of the cell cycle, while upregulated DEGs were enriched in ribosomal components, fatty acid metabolism (e.g. *CPT2* and *ACADL*), and oxidative phosphorylation (e.g. *NDUFA6*, *COX4I1*, *COX15*, and *ATP6V0B*), indicating enhanced energy metabolism and protein synthesis in tetraploids. Organ-specific analyses revealed sustained upregulation of energy metabolism and ribosomal genes in the lung, despite overall transcriptional downregulation (supplementary fig. S9, Supplementary Material online). Additionally, energy metabolism was also transcriptionally upregulated in the kidney and skin of tetraploids, suggesting increased energy demands at 25 °C, consistent with metabolic measurement.

**Fig. 6.**
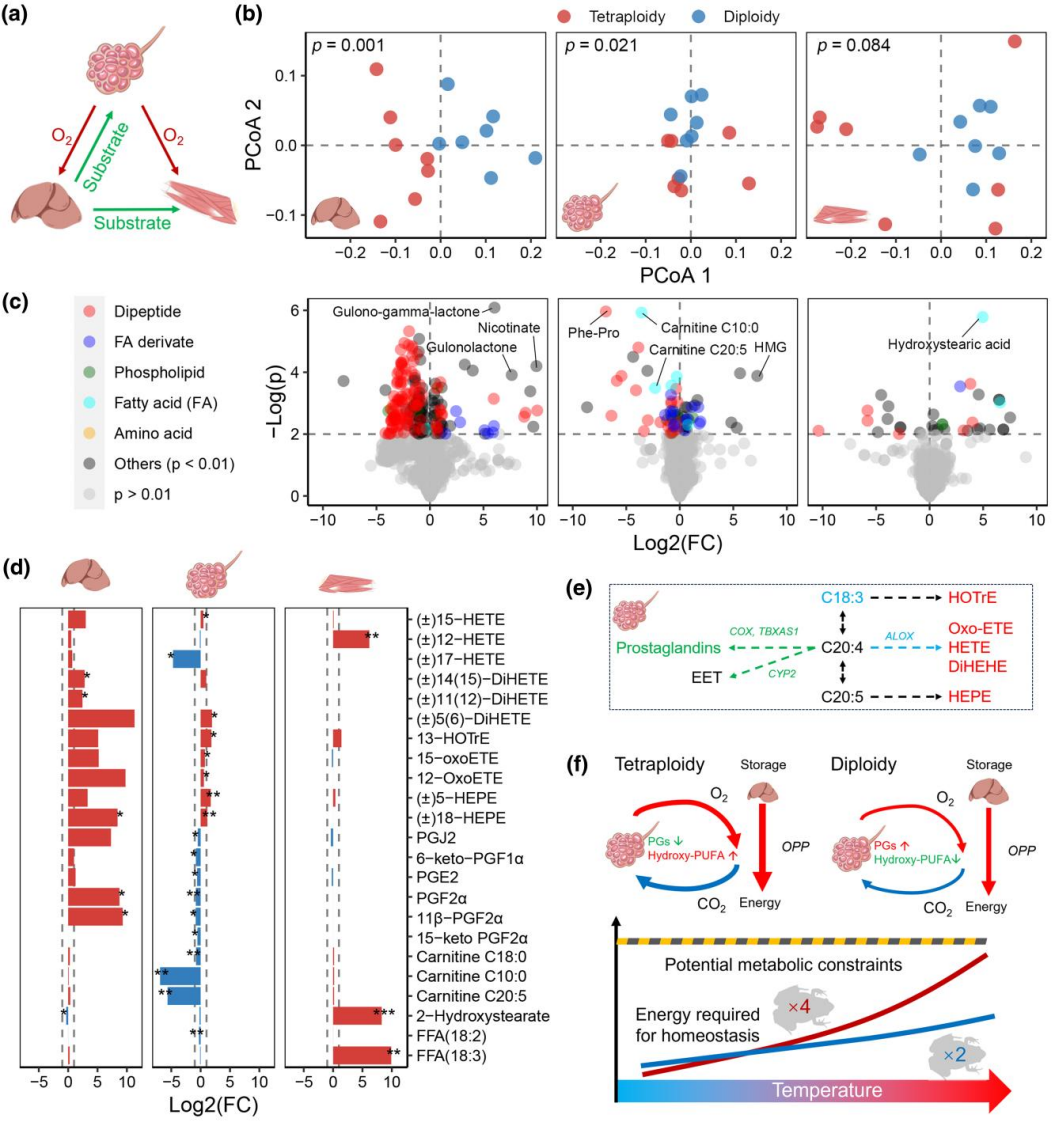
Metabolic differences in the lung, liver, and hindlimb muscle between diploid and tetraploid *M. fissipes* at room temperature (25 ± 0.5 °C). a) A schematic diagram describing the significance of these three organs in the metabolism. b) PCoA scatter plots presenting the dissimilarity in organ metabolome between diploid and tetraploid individuals. The data were analyzed with PERMANOVA. c) Volcano plots presenting the differential metabolites between diploid and tetraploid individuals (tetraploidy vs. diploidy). d) The variation pattern of differential fatty acids and derivates between diploid and tetraploid individuals (tetraploidy vs. diploidy). The data were analyzed using Student's *t*-test and BH correction: *, *P* < 0.05; **, *P* < 0.01; ***, *P* < 0.001. e) Combined transcriptomic and metabolomic analyses of polyunsaturated fatty acid metabolism. The red, green, blue, and black colors indicate increased/upregulated, decreased/downregulated, unchanged, and undetected levels in tetraploid individuals, respectively (at threshold of *P* < 0.05). f) A schematic diagram depicting the metabolic differences between diploid and tetraploid individuals. The width of the arrows denotes the estimated activity of the metabolic processes at 25 °C, based on a combination of physiological, transcriptomic, and metabolic data. In brief, the tetraploid demonstrates a higher metabolic consumption under elevated temperatures, making it more susceptible to constraints in oxygen capacity or resource availability.

To gain molecular insight into the divergent thermal metabolic properties, we analyzed the metabolomes of three key metabolic organs in males maintained at 25 °C: the lung (oxygen supplier), liver (substrate supplier), and muscle (consumer) (Fig. 6a). The liver exhibited the most pronounced metabolomic differences between diploids and tetraploids, followed by the lung (at threshold of *P* < 0.05, PERMANOVA; Fig. 6b). Differential analyses highlighted a significant reduction in dipeptides, an important amino acid storage used as an energy substrate in amphibians during starvation (Zhu et al. 2019; Zhu et al. 2020), in both the liver and lung of tetraploids (Fig. 6c). Additionally, major differences were observed in active FFA derivatives, including hydroxy-polyunsaturated fatty acids (e.g. HEPE, HETE, diHETE, and oxo-ETE) and prostaglandins (Fig. 6c and d), which regulate pulmonary vascular pressure and bronchial ventilation (Said et al. 1974; Lock et al. 1980; Fuller et al. 2015). In tetraploids, prostaglandin levels in the lung decreased with downregulation of key synthetic genes, while most hydroxy-polyunsaturated fatty acids increased without significant changes in their synthetic gene expression (Fig. 6e). This distinct pattern suggests that polyploidy involves adaptive adjustments in lung metabolites. Together, tetraploids showed reduced dipeptide storage and altered pulmonary regulatory compounds, likely reflecting the molecular consequences of their higher metabolic demands at elevated temperatures (Fig. 6f).

### Ecological Differentiation Between Diploids and Tetraploids

**Fig. 7.**
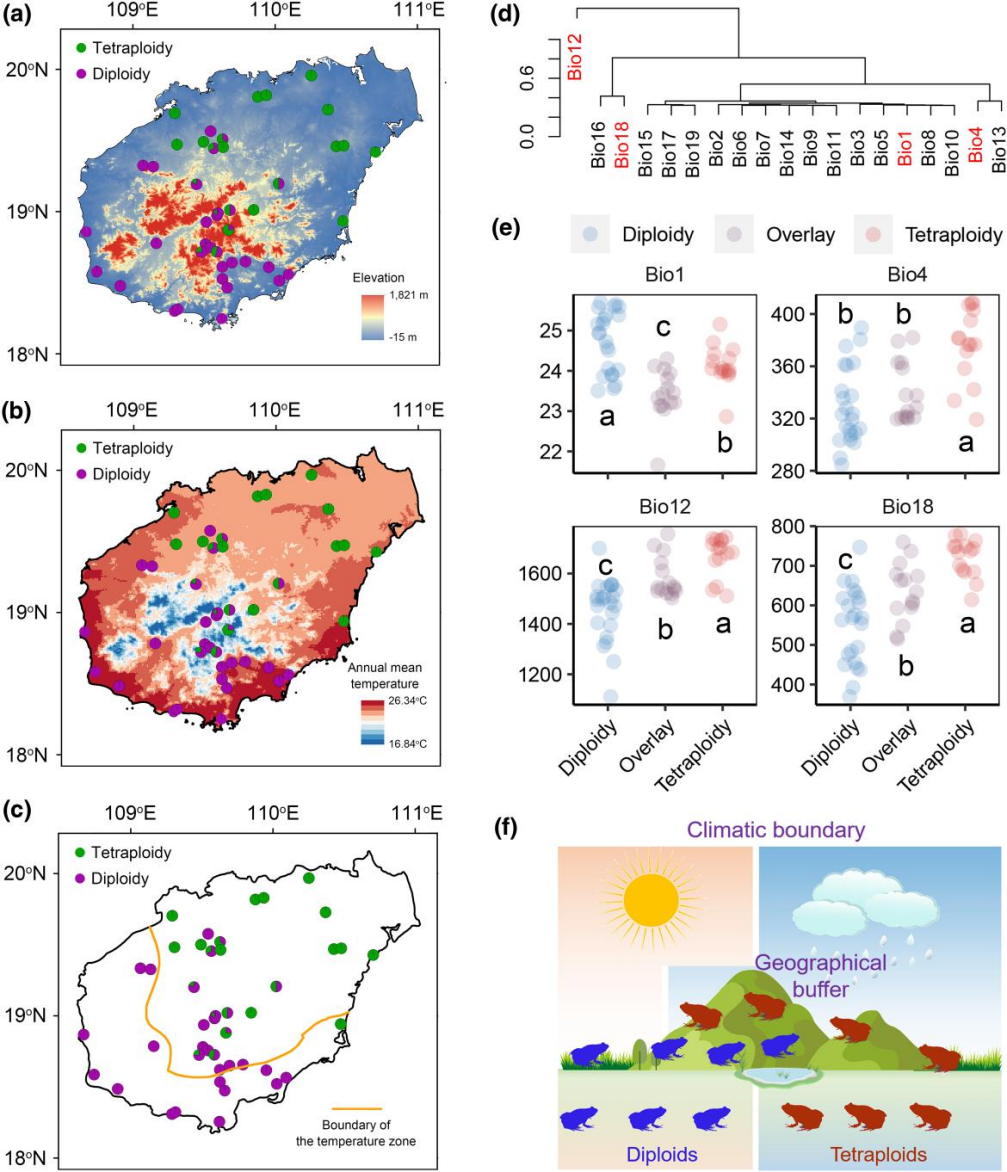
Ecological differentiation of diploid and tetraploid *M. fissipes*. a) The relationship between the distribution area and topography. b) The relationship between the distribution area and annual mean temperature. c) The relationship between the distribution area and boundary of the temperature zone. The boundary was constructed according to Che et al. (2014). d) Clustering of the climatic factors. The factors in red color were selected to substitute for the overall characteristics of the climatic factors. e) Variations in representative climatic factors with the distribution areas. The data were analyzed using one-way ANOVA. Different letters denote significant differences between groups. f) A schematic diagram depicting the potential boundary-separating effect of climate on diploid and tetraploid individuals, as well as the buffering effect of mountain ranges on these climate boundaries.

The distinct geographical distributions of diploid and tetraploid individuals suggest potential environmental segregation. Despite the presence of mountains dividing the island, they do not completely separate the northeast and southwest regions, as both diploids and tetraploids coexist in these mountainous areas (Fig. 7a), ruling out geographical isolation. Instead, tetraploid distribution closely aligns with the temperature zone boundary on Hainan Island (Fig. 7b and c). Additionally, tetraploids locations exhibit significantly lower annual mean temperatures (bio1), higher temperature seasonality (bio4), greater annual precipitation (bio12), and increased precipitation during the warmest quarter (bio18) (Fig. 7c and d). These findings point to climatic differentiation between diploids and tetraploids, driven primary by temperature. The mountain regions, with their vertical climatic gradient, likely act as a climatic buffer, enabling the coexistence of both types (Fig. 7f).

## Discussion

In this study, we identified the tetraploid *M. fissipes* on Hainan Island. These tetraploid individuals exhibited significant differences in male advertisement calls with their diploid counterparts, indicating the presence of pre-mating isolation in natural populations. Additionally, our laboratory experiments revealed a significantly reduced spawning rate when diploid females mated with tetraploid males, suggesting post-mating isolation. Although no reduction in spawning rate was observed when diploid males mated with tetraploid females, and F1 triploid offspring grew normally, hybrid sterility was evident, indicating post-zygotic isolation between diploids and tetraploids. These findings suggest that tetraploid and diploid *M. fissipes* represent distinct species.

Regarding polyploid formation, both karyotype and genomic data support the hypothesis that this polyploid originated via autopolyploidization, similar to the polyploidy observed in the amphibian *Dryophytes versicolor* (Wasserman 1970; Bogart and Wasserman 1972; Bogart et al. 2020). The tetraploid *M. fissipes* genome represents the first documented autotetraploid genome in an amphibian species. Autotetraploids can arise through either direct duplication of diploid genomes or a “triploid bridge” mechanism, which involves a maternal parent with facultative parthenogenesis (Ralin and Selander 1979). Given the absence of triploid *M. fissipes* in the wild and the reproductive isolation between diploids and tetraploids, it is more likely that tetraploid *M. fissipes* originated via direct duplication of diploid genomes.

Consistent with our hypothesis, polyploidization has induced significant changes in *M. fissipes* across multiple biological levels, with the most prominent being an increase in body size. Although polyploidy is typically associated with increased cell size (Orr-Weaver 2015), it often does not result in larger body size in vertebrates due to a compensatory reduction in cell number (Fankhauser 1945; Henery et al. 1992; Cadart et al. 2023). For example, artificially induced triploid *X. laevis* exhibits growth rates similar to or even lower than those of diploids throughout development (Cadart et al. 2023). In contrast, tetraploid *M. fissipes* demonstrates a distinct size advantage that becomes evident during the tadpole stage. This may represent a rare exception among vertebrates, with the underlying mechanism for this discrepancy yet to be elucidated. Given that *M. fissipes* tadpoles were not overfed and both diploids and tetraploids were maintained under identical conditions, the larger size of tetraploid tadpoles suggests more efficient resource conversion. Two factors may explain this phenomenon. First, tetraploids, both in the tadpole and adult stages, showed reduced cell cycle activity compared to diploids. This reduction can be attributed to the larger cell size in tetraploids, which decreases the number of cell divisions required to achieve the same body mass. Fewer cell divisions mean a shorter duration spent in the mitotic phase, during which transcription is silenced and chromosomes are condensed (Zaret 2014). Additionally, mitosis is energetically demanding, involving processes such as the synthesis and degradation of cyclins and chromosome segregation (Murray and Kirschner 1989; Salazar-Roa and Malumbres 2017). By reducing the frequency of these energy-intensive divisions, tetraploids may achieve greater time and energy efficiency during growth, contributing to their larger size. Second, tetraploids exhibit distinct metabolic profiles compared to diploids, though the precise link between chromosomal duplication and these metabolic changes remains unclear. Specifically, tetraploids likely utilize alternative glucose metabolism pathways more effectively, such as the pentose phosphate pathway and the uronic acid pathway. These pathways generate critical substrates like NADPH, which are essential for biosynthetic and anabolic processes (Rahman and Hasan 2014). Coupled with potential upregulation of energy metabolism (i.e. TCA cycle) in tetraploids, these metabolic shifts likely contribute to their superior resource utilization efficiency. In addition to changes in overall body size, polyploidization in *M. fissipes* induced shifts in organ allometry, resulting in altered scaling relationships between organs and body mass. Notably, the slope of the relationship between reproductive organ size and body weight was significantly higher in tetraploid individuals, particularly in females. This suggests that tetraploids may allocate more resources to reproduction, potentially reflecting an increased investment in reproductive fitness.

Tetraploid *M. fissipes* males showed increased metabolic rate at higher temperatures (e.g. 28 to 34 °C) compared to the diploids, which was supported by the transcriptional upregulation of energy metabolism across organs at 25 °C, a relatively high temperature. This variation trend was opposite to those observed in artificially induced polyploids, which showed decreased metabolic rate (Atkins and Benfey 2008; Polymeropoulos et al. 2014; Hermaniuk et al. 2017, 2021; Cadart et al. 2023). Although the energy budget for maintaining cell membranes and ionic gradients likely decreased due to reduced cellular surface area (Cadart et al. 2023), tetraploid *M. fissipes* may have increased energy requirements in other areas due to physiological adjustments from long-term evolution for environmental adaptation. These can be resulted from variations in the relative size of energy-intensive organs, relative proportions of energy-intensive cells, and metabolic reorganization. Additionally, this discrepancy may arise from the inherent distinctions in polyploidization between *M. fissipes* and other species. For instance, polyploidization in *M. fissipes* leads to a significant increase in body size, whereas in other vertebrates, it usually results in minimal changes in body size.

The biological consequences of polyploidization in *M. fissipes* do not provide absolute advantages or disadvantages in competition with diploid individuals or environmental adaptability. Larger body size offers benefits such as improved resource competition and reduced predation risk (Shine 1988; Blanckenhorn 2000), and the enhanced jumping ability and increased reproductive investment observed in tetraploids may further strengthen their competitive edge over diploids. However, these advantages come with tradeoffs. The significantly higher energy demands of tetraploids at elevated temperatures suggest they may encounter greater resource challenges in hot environments, a hypothesis supported by the markedly reduced hepatic dipeptide content in tetraploids (Fig. 6f). Beyond nutrient demands, oxygen availability is another critical factor for sustaining metabolism. Notably, tetraploid males exhibit relatively smaller cardiac size, while tetraploid females display a significantly lower scaling relationship between heart and lung size relative to body weight compared to diploids. Given that relative cardiac size is a key predictor of maximal oxygen consumption in animals (Hammond et al. 1992; La Gerche et al. 2012), these findings suggest that tetraploid adults are more likely face limitations in oxygenation capacity at higher temperatures. This may be supported by our metabolomic data, which reveals significant variations in pulmonary regulatory compounds in tetraploid lungs at 25 °C, suggesting that pulmonary function is likely a key factor in the adaptive regulation of polyploids. Moreover, the reduced surface area-to-volume ratio resulting from larger cell size in tetraploids may further exacerbate their oxygen exchange limitations under thermal stress. Therefore, the survival advantage conferred by polyploidization in *M. fissipes* is dependent on environmental conditions.

It is intriguing to explore whether and how polyploidization drive ecological differentiations in species. In plants, eco-physiological differentiation, along with changes in environmental adaptability and distribution associated with polyploidy, have been extensively investigated (Buggs and Pannell 2007; Baniaga et al. 2020), providing a mechanistic understanding of species distribution patterns. However, research on vertebrates remains limited. In this study, we demonstrate that polyploidy-related physiological changes can help explain the distribution patterns of diploid and tetraploid *M. fissipes*. Specifically, we found a significant association between the distribution of diploids and tetraploids and climate factors. Tetraploid individuals are more prevalent in the northeastern regions with lower average annual temperatures, while diploids are predominantly found in the southwestern regions with higher temperatures. This climatic differentiation can be attributed to the tradeoffs in tetraploid physiological performance: advantages in body size, reproduction, and locomotion, but with higher energy demands and potential metabolic constraints at elevated temperatures. Our findings offer insights into how polyploidy influences morphology, physiology, and metabolism, and how these differences may impact biological performance, ultimately shaping the geographic and climatic patterns observed between diploids and tetraploids, improving our understanding of the role of polyploidy in species evolution and diversification.

The physiological differences between diploid and tetraploid *M. fissipes* are meaningful for predicting their future fates. For instance, as global warming progresses (Scheffers et al. 2016), the distribution balance between diploids and tetraploids may be disrupted. While climate change could negatively affect both, tetraploids, with their higher metabolic demands at elevated temperatures, may face more severe consequences. This could include a shift in their primary habitats to regions with lower average annual temperatures. Given that the island limits northward migration, the mountainous central areas of the island, the overlapping regions of diploids and tetraploids, may serve as refuges for tetraploids. It means that the tetraploid *M. fissipes* in this region may warrant special attention and conservation. Collectively, polyploidy provides a robust model for investigating how genetic variation drives physiological differences, resulting in shifts in environmental adaptability and ecological differentiation. These findings offer not only substantial theoretical value in evolutionary biology but also a mechanistic framework for predicting the impacts of environmental changes on animal populations, with important implications for species conservation and management.

## Conclusion

In this study, we identified an autotetraploid *M. fissipes* on Hainan Island, likely originating from direct genome duplication of a diploid ancestor. Its reproductive isolation from diploid *M. fissipes* suggests it represents a distinct species. While tetraploid and diploid *M. fissipes* coexist on the island, their distribution areas exhibit significant geographical and climatic differentiation, with tetraploids predominantly occupying the cooler and wetter northeastern regions. Tetraploids exhibit greater body size but are not simply scaled-up versions of diploids. They differ from diploids in physiological traits, organ allometry, gene transcriptional profiles, and metabolic patterns. The biological consequences of polyploidization offer neither absolute advantages nor disadvantages in competition or environmental adaptability. On one hand, tetraploids display larger body size, superior jumping ability, and greater reproductive investment, potentially conferring a competitive edge over diploids. On the other hand, tetraploids exhibit higher metabolic demands at elevated temperatures, lower energy storage in the liver and altered pulmonary regulatory compounds in the lung. Combined with their relatively smaller heart size, these traits suggest that tetraploids are more likely to face metabolic constraints related to substrate and oxygen availability under heat stress, limiting their capacity for long-term metabolic maintenance in warmer environments. This tradeoff in physiological performance likely underlies the geographical separation of diploids and tetraploids, providing an example of how polyploids ecologically differentiate and interact with closely related diploids. These findings enhance our understanding of polyploid fate and the role of polyploidization in vertebrate evolution and biodiversity. Further research could further explore the links between chromosomal duplication, organ allometry, and metabolic divergence between diploid and tetraploid *M. fissipes*.

## Materials and Methods

### Sampling Collection and Indoor Culture

*Microhyla fissipes* individuals were collected from Hainan Island of China from 2019 to 2021 (detailed in supplementary table S1, Supplementary Material online). The sex of adult individuals was determined by identifying swollen testes and spermatic ducts in males, and the presence of oocytes in females. The frogs were kept in plastic containers (30 × 20 × 15 cm), with 2 to 4 individuals per container, at a constant temperature of 25 ± 0.5 °C, and a 12-h light/dark cycle. They were fed fruit flies (*Drosophila sp.*) once daily, with an average of five flies per frog per day. Indoor breeding followed our previously established standard procedures for artificially inducing mating and spawning (Wang et al. 2017). The fertilized egg clutches were transferred to new containers (42 × 30 × 10 m), with a water depth of 5 cm. The hatched tadpoles were fed a solution of boiled chicken egg yolk once a day for 2 d. The tadpoles were then fed spirulina powder (China National Salt Industry Corporation) once a day, and the water was replaced every 2 days. The developmental stages of tadpoles were identified according to the staging table reported by Wang et al. (2017). All the procedures applied for this study were approved by the Institutional Ethics Committee of Animal Ethical and Welfare Committee of Chengdu Institute of Biology, Chinese Academy of Sciences (permit: CIB20190201), and all the methods were carried out in accordance with the Code of Practice for the Care and Handling of animal guidelines. This study is reported in compliance with the ARRIVE guidelines. We have complied with all relevant ethical regulations for animal use.

### Morphological Analyses

The morphological traits of adult frogs were measured, and their ploidy was determined with karyotype. These included whole-body weight, snout-vent length (SVL), head length, head width, interorbital space, and hindlimb length. The mass of each individual was measured using a digital scale to the nearest 0.01 g. The length was measured with a dial caliper to the nearest 0.01 mm. The data were provided in supplementary table S1, Supplementary Material online.

To evaluate the growth performance of diploid and tetraploid tadpoles, three diploid and tetraploid populations (tadpoles from the same container treated as a population) were recorded for their whole-body length and developmental duration after they reached to stage 28 (Gosner 1960). Each population included 30 individuals. When over half of the individuals in a container reach the next developmental stage, the entire container is considered to have advanced (supplementary table S2, Supplementary Material online). Tadpoles are then randomly selected (*n* > 4 for each population at each stage) for length measurement (nearest to 0.01 mm) under a microscope (supplementary table S3, Supplementary Material online). Measurements are taken only on the day the tadpoles reach a specific stage. Upon reaching stage 45 and completing metamorphosis, the whole-body weight (nearest to 0.1 mg) was also measured (supplementary table S4, Supplementary Material online).

### Karyotypic Identification

The bone marrow chromosome specimens were prepared following the experimental procedures of Wu (1982) with modifications. A 0.1% colchicine solution (prepared with 0.65% physiological saline) was injected subcutaneously into live frogs at a dose of 10 μL/g body weight. After the first injection, frogs were kept at 24 °C for 12 h before the second injection. The second injection was administered into the frog’s abdominal cavity using the same colchicine solution and dosage. Then, frogs were euthanized with ether, and the tibia and femur bones were removed. Bones were transferred to glass slides, immersed in 0.4% KCl hypotonic solution to release bone marrow. Insoluble impurities were removed, and hypotonic solution was added to cover the glass slides. Glass slides were placed in large glass dishes, supported with glass rods, covered, and left undisturbed for 30 min. After hypotonic treatment, a mixed fixing solution (ethanol:acetic acid:water = 1:2:3) was slowly added to the dishes without touching the glass slides. The dishes were covered and sealed, maintained at 24 to 28 °C for 120 min. After fixation, the bottom of the dishes was aspirated, replaced with anhydrous ethanol for a second fixation for 30 min. Glass slides were tilted to drain the hypotonic solution, and then fixed with a solution of ethanol and acetic acid (1:2) poured from the top. This process was repeated three to four times before air-drying. The chromosomes were then stained with Giemsa (10% for 30 min), and photographed using an Optec B302 microscope equipped with a Sony ICX285A CCD camera. Chromosomal measurements were conducted using ImageView software and analyzed based on Levan's criterion (Levan et al. 1964). The parameters of chromosomal pairs or tetrapartites were detailed in supplementary table S5, Supplementary Material online. Along with karyotypic identification, the whole-body and organ weight were measured nearest to 0.1 mg (supplementary table S6, Supplementary Material online). Note that gonadal weight was excluded from the total body weight in our analysis of the relationship between organ weight and body weight. This is because gonadal weight is highly variable, fluctuating significantly both across individuals and over time. Removing the gonads provides a more accurate measure of an individual's true size. Additionally, gonads constitute a large proportion of body weight, with considerable individual variation, which can introduce significant random error and obscure the true relationship between organ and body weight.

### Bioacoustics

We recorded advertisement calls of 8 males of *M. fissipes* from Wuzhishan City, an overlapping distribution area of diploid and tetraploid individuals. Each individual was recorded at a distance between 10 and 20 cm using a Marantz PMD661 digital voice recorder with an external microphone (Sennheiser ME66/K6), and 15 calls were recorded for each individual. The sound files in wave format were resampled at 48 kHz with sampling depth 24 bits. The sonograms and waveforms were generated by R package seewave (Sueur et al. 2008). Adobe Audition 3.0 (USA, San Jose, CA) was used to extract and analyze the acoustic characteristics of calls. The acoustic characteristics included in the analysis are as follows: call duration, the time from the start of the first pulse to the end of the last pulse in a single call; call interval, the time from the end of one call to the start of the next call; pulse duration, the time from the start to the end of a single pulse; pulse interval, the time from the end of one pulse to the start of the next pulse; pulses per call, the total number of pulses within a single call; dominant frequency, the frequency with the highest energy distribution within the call. FactoMineR (Husson et al. 2013) and factoextra (Kassambara 2016) packages were used to perform Principal component analysis (PCA).

### Measurement of Physiological Parameters

The diploid and tetraploid adults were collected from Sanya (Southeast) and Haikou (Northeast), in Hainan Island. All the filed-collected individuals were acclimated to 25 °C indoors for at least 2 weeks.

#### Jumping Performance

The measurement of jumping performance was conducted indoors within an enclosed space. The floor and walls of the experimental area were pre-covered with white background panels, and a fixed measuring tape was installed. The entire environment was maintained free from noise and human movement disturbances, and cameras were set up to record the entire process. During the experiment, each individual was carefully placed in the center of the site. From a distance of ∼2 m, a plastic rod was used to tap the posterior end of each frog, stimulating them to jump. This procedure was repeated for seven times for each individual. After the frogs completed their jumps, the distance of each jump was measured based on the footprints left on the background panels. Following the jumping trials, the frogs underwent sex identification, weight measurement, SVL measurement, and karyotype identification. The measurement included 120 individuals (*n* = 30 per gender per ploidy) (supplementary table S7, Supplementary Material online).

#### Metabolic Rate

We used a closed respirometry system to measure the basic metabolic rate (oxygen consumption rate) of adult males. The oxygen level was measured using a FireSting-O_2_ oxygen meters (Pyro Science, Aachen, Germany). The respirometer has four detection channels, allowing three parallel measurements, and one blank control in the same batch. Each channel was linked to a measurement chamber, which was placed in a climate chamber (Shanghai Boxun Industrial BIC-250) to control the temperature. During the experiment, the frogs were allowed to acclimate to the measurement temperature for 30 min, and measurements began when the frogs were in a resting state, with the oxygen level detected every second. Variation in O_2_ were recorded using real-time curve graphs, and the curves of the experimental groups were adjusted using the values of black control. The slopes of the curves were calculated through linear fitting, and frogs were weighed after each round of measurement. The metabolic rate was measured at 16, 22, 28, and 34 °C sequentially. The metabolic rate of each individual was measured at all the four temperatures. Once the measurement at each temperature completed, the frog was put back to 25 °C for routine culture, and the interval between measurements at two different temperatures were 7 d for each individual.

The metabolic rate was calculated following the formula below (Zhu et al. 2016):


$$\mathrm{Metabolic}\;\mathrm{rate}=\frac{\mathrm{Slope}\times \mathrm{Pressure}\times \mathrm{Volume}}{\mathrm{Temperature}\times \mathrm{Weight}\;\times \;R}$$

where, metabolic rate is the consumption rate of O_2_, mol/s/g; slope is the curve slope O_2_ consumption (%/s); pressure is the air pressure in the closed-circuit system, 101,325 Pa; volume is the volume of the closed-circuit system, 0.0001 m^3^; temperature is the temperature of the closed-circuit system, K; and *R* is the ideal gas constant (8.314 J/mol/K); weight is the body weight of the frog (g). The data were provided in supplementary table S8, Supplementary Material online.

### Histological Analysis

The F1 adults of diploid, triploid (tetraploid females × diploid males) and tetraploid were euthanized using MS-222 (three individuals per gender per group). Their ovaries or spermaries were collected and preserved in 4% paraformaldehyde. The subsequent procedures included dehydration in an ethanol gradient, clearing with xylene, embedding in paraffin, and preparation of serial transverse sections (7 μm thickness). The dewaxed serial sections were stained with Delafield’s hematoxylin and counterstained with eosin (H & E staining). The histological sections were photographed using an Optec B302 microscope equipped with a Sony ICX285A CCD camera.

### Genome Sequencing and Assembly

Whole-genome sequencing was performed for diploid and tetraploid *M. fissipes* females. DNA was extracted from muscle using the SDS method. For diploid, the assembly of genome was accomplished by combining Illumina short reads and Pacbio long reads. Total 439.60 Gb Illumina reads were generated by Illumina Hiseq platform, and 351.43 Gb PacBio reads were obtained from Pacbio Sequel. For tetraploid, Illumina short reads and Nanopore long reads were used to assembly the genome. Total 472.02 Gb illumina reads were performed on Illumina Hiseq platform and 38.49 Gb Nanopore reads were obtained from PromethION. To achieve chromosome-level genome, Hi-C sequencing on an Illumina HiSeq X platform was employed for the assembly of both the diploid and tetraploid genomes.

The software NextDenovo (https://github.com/Nextomics/NextDenovo) was used to assemble the contig using default parameters. Then, the Illumina reads were aligned to the assembled genome using BWA with default parameters. The processed aligned files were used to polish the initial draft assembly with two rounds of NextPolish v. 1.0 (Hu et al. 2020). The contigs were assembled into chromosome-level scaffolds using Bowtie2 v2.3.2 (supplementary table S9, Supplementary Material online) (Langmead and Salzberg 2012). The completeness of the genome was assessed against vertebrate lineages using Benchmarking Universal Single-Copy Orthologs (BUSCO v. 3.0.2) (supplementary table S10, Supplementary Material online) (Simão et al. 2015).

For de novo prediction of repeats, we used RepeatModeler v1.0.8 (http://www.repeatmasker.org/RepeatModeler.html) software to build a de novo repeat library. RepeatMasker v. 3.3.0 (Chen 2004) was employed to construct the homology repeat library. Additionally, we used Tandem Repeats Finder (Benson 1999) to recognize all tandem repeat elements in the entire genome.

The gene prediction and functional annotation process incorporated multiple methods including homology-based prediction, de novo prediction, and transcriptome-based prediction. For the homology-based prediction, protein sequences from multiple species, including *X. laevis*, *X. tropicalis*, *L. leishanense*, *Aquarana catesbeiana*, *N. parkeri*, and *Microcaecilia unicolor*, were downloaded from public datasets (supplementary table S11, Supplementary Material online). These sequences were then aligned to the diploid and tetraploid *M. fissipes* genomes using TblastN (E-value ≤ 1e-5). The resulting BLAST hits were further aligned and refined using GeneWise (v2.4.1) for accurate spliced alignments. Three tools, namely Augustus (v2.7), GlimmerHMM (v3.02), and SNAP (version 2006-07-28), were utilized for de novo gene prediction against the repeat-masked genome sequences. The RNA-seq reads from multiple tissues were mapped to the genome assembly using Tophat (v2.1.0), and then Cufflinks (v2.1.1) assembled the transcripts into gene models based on this mapping information. The gene predictions obtained from the de novo approach, homology-based approach, and RNA-Seq-based evidence were merged to create a comprehensive consensus gene set using the software EVM (Haas et al. 2008).

To perform functional annotation, the predicted protein sequences were aligned against public databases (i.e. SwissProt, TrEMBLE, and KEGG) using BLASTP (E-value ≤ 1e-5). Additionally, protein motifs and domains were annotated by searching InterPro and Gene Ontology databases using InterProScan (v4.8).

### Comparative Genomic Analyses

Diploid and tetraploid *M. fissipes* were subjected to comparative genomic analyses alongside nine additional species, including *H. sapiens*, *M. musculus*, *G. gallus*, *A. carolinensis*, *L. leishanense*, *R. temporaria*, *N. parkeri*, *B. gargarizans*, and *X. tropicalis*, which were obtained from public databases (supplementary table S11, Supplementary Material online). In our comparative genomic analysis, the tetraploid genome was partitioned into two distinct subgenomes, designated as A and B. We assessed chromosome synteny between diploid and tetraploid *M. fissipes* (subgenomes A and B) by conducting all-to-all BLASTP searches of protein sequences, employing an E-value threshold of 1e-5. Collinear blocks, each encompassing a minimum of 10 genes (-s 10) and allowing for a maximum of 25 gaps (-m 25) between two adjacent orthologs within a block, were delineated using MCScanX (Wang et al. 2012).

The identification of single-copy gene families across the 11 genomes was facilitated by OrthoFinder v2.5.4 (Emms and Kelly 2019). Subsequent alignment of these gene families was performed using MUSCLE (Edgar 2004). The aligned coding sequences were concatenated to form super-genes for each species. A species tree was then inferred using RAxML (Stamatakis 2014), based on the super-genes of each species. Divergence times for the phylogenetic tree were estimated with the MCMCTREE program within PAML v4.7 software (Yang 2024). Calibration points for the analysis were sourced from the TimeTree database (http://www.timetree.org/).

Gene family expansion or contraction was analyzed using CAFÉ v4.2.1 (De Bie et al. 2006), based on the aforementioned phylogenetic tree. The cluster size at each branch was compared with that of the ancestral node. Significance of expansion or contraction was determined by calculating *P*-values using the Viterbi algorithm under a hidden Markov model framework, with *P* < 0.05 indicating statistically significant changes.

### Transcriptional Analyses

Tissue-specific transcriptomics were performed for the adult brain, heart, liver, lung, kidney, skin, muscle, ovary, and testis of diploid and tetraploid individuals (*n* = 4 per tissue per gender per ploidy). Whole-tissue transcriptomics were performed for tadpoles at Gosner stage 39 and 41 (pro-metamorphosis), stage 43 (metamorphic climax), and stage 45 (completion of metamorphosis) (Gosner 1960). Four samples were prepared for each phase as biological replications (*n* = 4 each tissue per ploidy per gender in adults; *n* = 4 each stage per ploidy). After dissection, all the samples were stored at −80 °C for further analysis.

The extraction of total RNA was performed using the TRIzol protocol (Life Technologies Corp., Carlsbad, CA, USA). For each sample, 1 μg of RNA was utilized for library construction using the NEBNext®Ultra™ RNA Library Prep Kit for Illumina® (NEB, Ipswich, MA, USA). Sequencing was carried out on an Illumina Hiseq 2000 platform by Biomarker Technologies Co., Ltd. (Rohnert Park, CA, USA), and paired-end reads were generated.

To mitigate the impact of varying gene duplicate numbers between diploid and tetraploid genomes, clean data from both diploid and tetraploid individuals were mapped to the diploid *M. fissipes* genome using Subread (v2.0.1) (Liao et al. 2013). In subsequent analyses, transcripts from genes present in multiple copies within the tetraploid genome were pooled for analysis, without distinguishing their subgenomic origin. This approach treats the expression levels as the combined sum of both subgenomes, rather than calculating expression separately for the two sets of chromosomes in the tetraploid genome. Differentially expressed genes (DEGs) were identified using DESeq2 (Love et al. 2014), with stringent thresholds of fold change > 2 (|log2Fold Change| > 1) and a q-value < 0.05, following Benjamini–Hochberg correction for multiple testing. Gene expression levels were normalized to transcripts per million (TPM) mapped reads using custom R scripts. This analytical pipeline, incorporating both DESeq2 normalization and TPM quantification, effectively neutralized biases arising from differences in sequencing depth and gene duplicate numbers.

### Untargeted Metabolomics

The lung, muscle, liver of adults, and entire tadpole of stage 39 were collected for metabolic profiles (*n* = 7 per tissue or individuals per ploidy). To extract the metabolites, 100 mg of tissue powder was ground in liquid nitrogen and then subjected to extraction using a mixture of methanol, acetonitrile, and water in a 2:2:1 (v/v) ratio. The extraction process involved ultrasonication for 30 min, repeated twice, followed by incubation at −20 °C for 1 h. Subsequently, the samples were centrifuged at 12,000 × *g* for 15 min at 4 °C. The resulting supernatants were transferred to new tubes and freeze-dried. Prior to analysis, the samples were reconstituted in 100 μL of a 1:1 (v/v) mixture of acetonitrile and water. The analysis was performed using liquid chromatography (Agilent 1290 Infinity LC, USA) coupled with quadrupole-time-of-flight mass spectrometry (Triple TOF 5600+, AB SCIEX, USA). The chromatographic analyses were performed by Shanghai Bioprofile Technology Company Ltd (China). To identify the metabolites, a standard library of MS/MS spectra constructed by Shanghai Bioprofile Technology Company Ltd was queried. The relative abundances/concentrations of the metabolites were represented by the ion intensities of their molecular ion peaks (supplementary tables S12 and S13, Supplementary Material online).

### Climatic Factor Analyses

The 19 climatic factors (Bio1−19) of the collection sites (supplementary table S1, Supplementary Material online) were obtained from WorldClim database (http://www.worldclim.org). The 19 climatic factors were clustered using *hclust* function (method = “*complete*”) in R (R Core Team 2021). The representative factors of each cluster were selected for subsequent differential analyses. The graphs were generated using ArcGIS 10.2 (ESRI, Redlands, CA, USA). The temperature zone and boundary were constructed according to Che et al. (2014).

### Statistical Analysis

Statistical analyses were conducted using SPSS v25.0 (SPSS Inc., Chicago, USA). Kolmogorov–Smirnov tests were employed to assess the departure of data from a normal distribution. Morphological data for each gender were analyzed using ANCOVA, with ploidy as the fixed factor and body weight or SVL as covariates. Wilcoxon tests were utilized to compare advertisement call characteristics between diploid and tetraploid individuals. Spawning rates were assessed using χ^2^ tests. Growth and developmental data were subjected to ANCOVA, with ploidy as the fixed factor and Gosner stage as a covariate. Tadpole body weight at stage 45 was examined using Student's *t*-test. Organ allometry differences between diploids and tetraploids were evaluated using ANCOVA, with ploidy as the fixed factor and body weight as a covariate. Jumping performance was analyzed using linear mixed models, incorporating ploidy and gender as fixed factors, body weight as a covariate, and individuals as a random factor. Due to a significant interaction between gender and ploidy, data for each gender were independently analyzed using ANCOVA, with ploidy as a fixed factor, body weight as a covariate, and individuals as a random factor. Metabolic rate was assessed using linear mixed models. When considering the effects of body weight, measuring temperature and ploidy were included as fixed factors, body weight as a covariate, and individuals as a random factor. When considering temperature effects, ploidy was treated as a fixed factor, measuring temperature as a covariate, and individuals as a random factor. In all models, interactions between factors and covariates were initially included, and insignificant interactive effects were removed in the final models. For multivariate analyses of transcriptomic and metabolic data, the expression matrix was converted to a Bray-Curtis distance matrix and analyzed using permutational multivariate analysis of variance (PERMANOVA, mutations = 9,999) (Dixon 2003). Principal coordinates analyses (PCoA) were conducted to illustrate dissimilarities between samples. Subsequent differential analyses on transcriptomic and metabolic data were based on Student's *t*-tests with Benjamini and Hochberg corrections. Gene enrichment analysis was performed using KOBAS 3.0 (Bu et al. 2021). Differences in climatic factors were assessed using one-way ANOVA followed by S-N-K post-hoc tests. Networks were visualized using Cytoscape 3.5.0. Other graphs were generated using GraphPad Prism 5 or ggplot2, an R package (Wickham 2009). The illustrations were created using MedPeer (medpeer.cn).

## Supplementary Material

msaf037_Supplementary_Data

## Data Availability

The genome assembly has been submitted to the China National GeneBank DataBase (CNGBdb, https://db.cngb.org/) under accession number CNP0005883, with assembly number CNA0141905 and CNA0141906 respectively. And the transcriptomics sequencing data from this study have been deposited at NCBI Sequence Read Archive (https://www.ncbi.nlm.nih.gov/) under accession number PRJNA1127044.
