# Supplementary material for: Every Gain Comes With Loss: Ecological and Physiological Shifts Associated With Polyploidization in a Pygmy Frog
Source: Mol Biol Evol. 2025 Feb 7;42(2):msaf037. doi: 10.1093/molbev/msaf037 (PMC11840752; doi:10.1093/molbev/msaf037)
Supplement: msaf037_Supplementary_Data [file msaf037_supplementary_data.zip › Supplementary data 1.pdf]

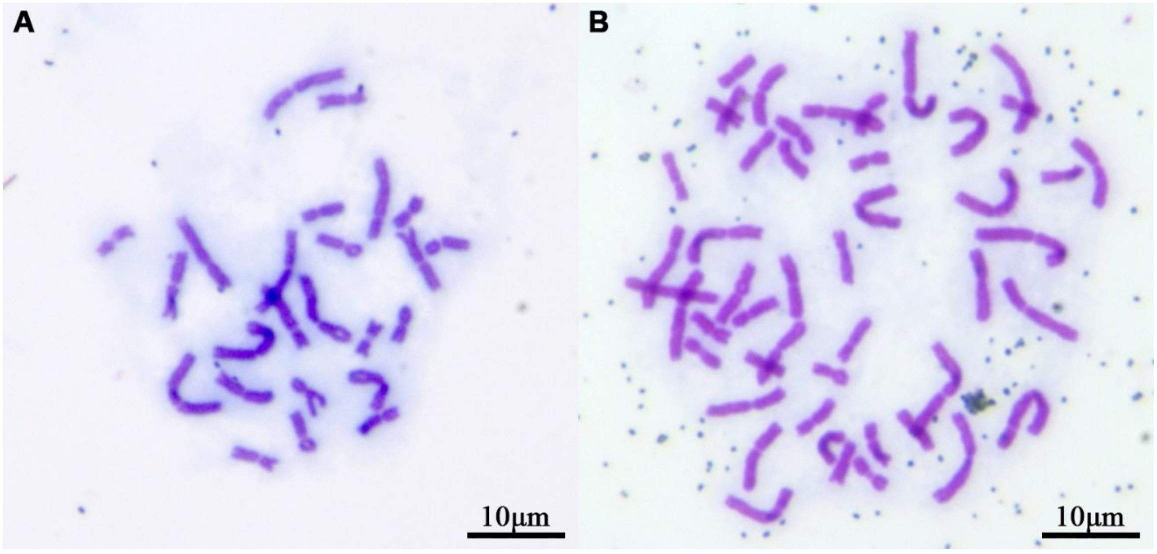

Figure S1 Karyotype of diploid (A) and tetraploid (B) *Microhyla fissipes* during mitotic metaphase.

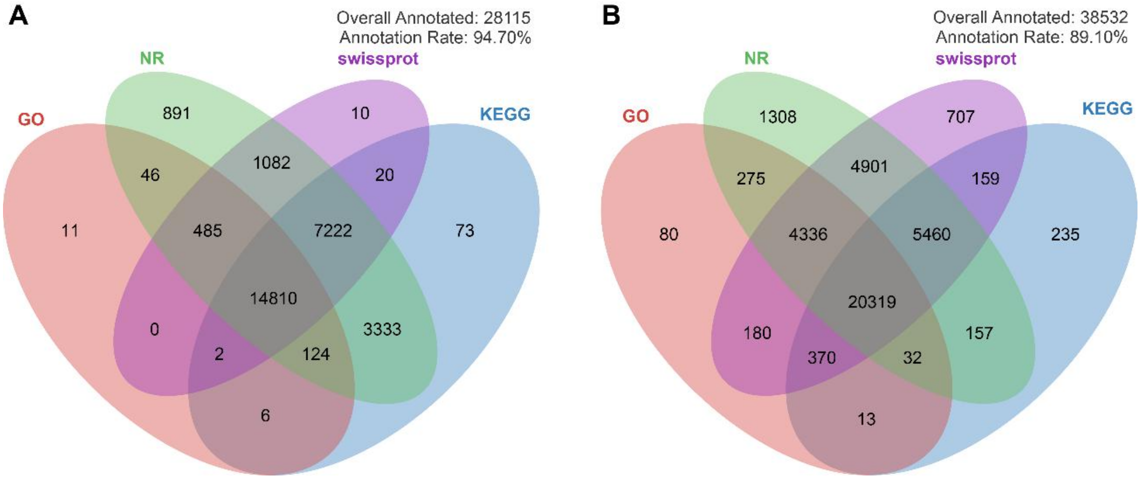

Figure S2 Functional annotation of genes in diploid and tetraploid genomes.

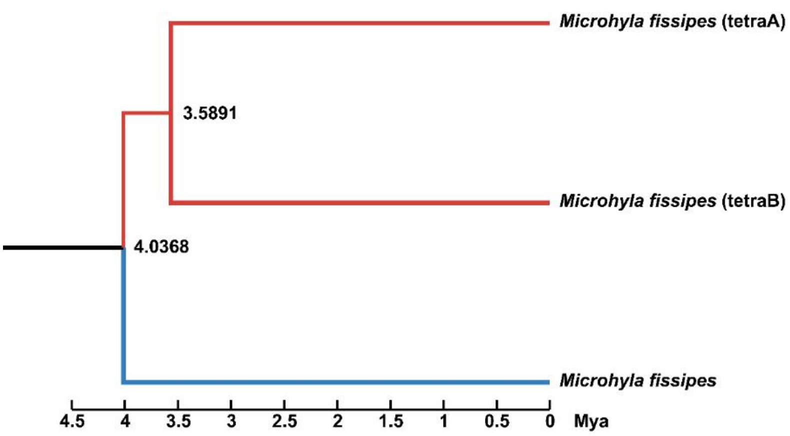

Figure S3 Estimation of divergence time of two sub-genomes of tetraploid.

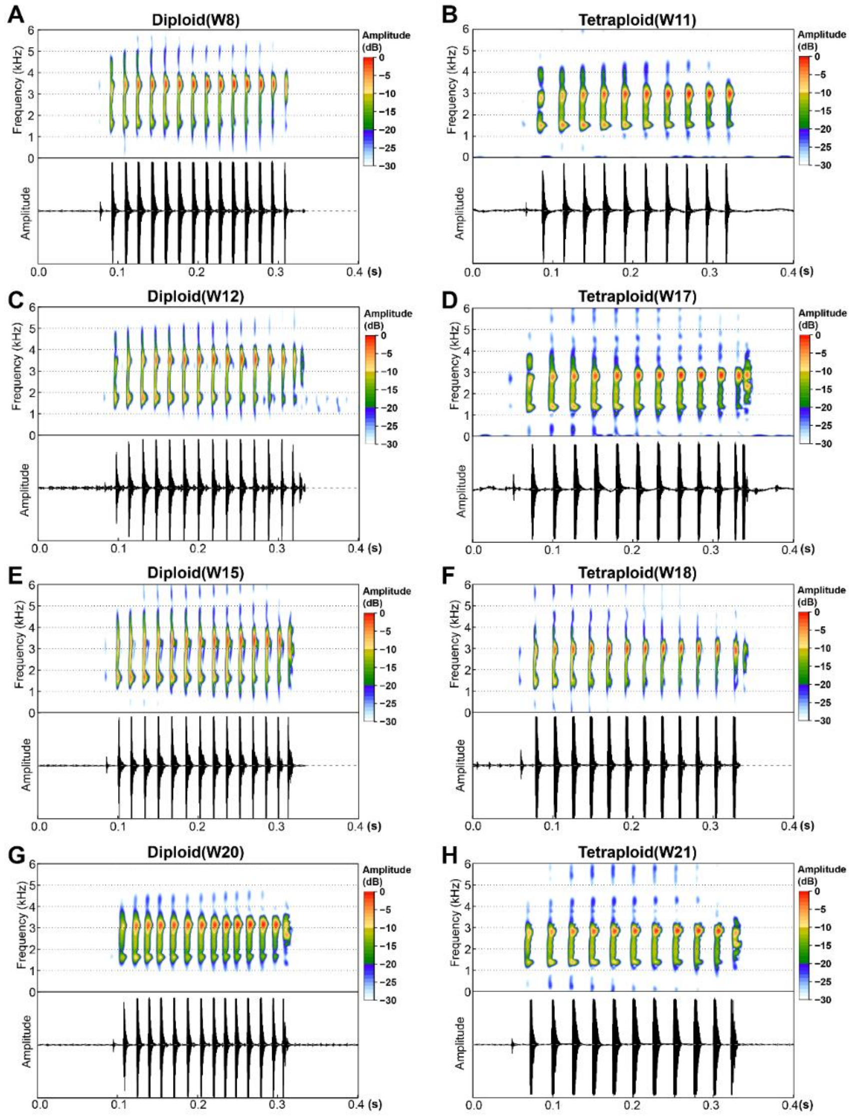

Figure S4 Advertisement calls of diploid and tetraploid *M. fissipes* from overlapping distribution sites on Hainan Island. (A, C, E, G) Visualization of advertisement calls of diploid individuals. (B, D, F, H) Visualization of advertisement calls of tetraploid individuals.

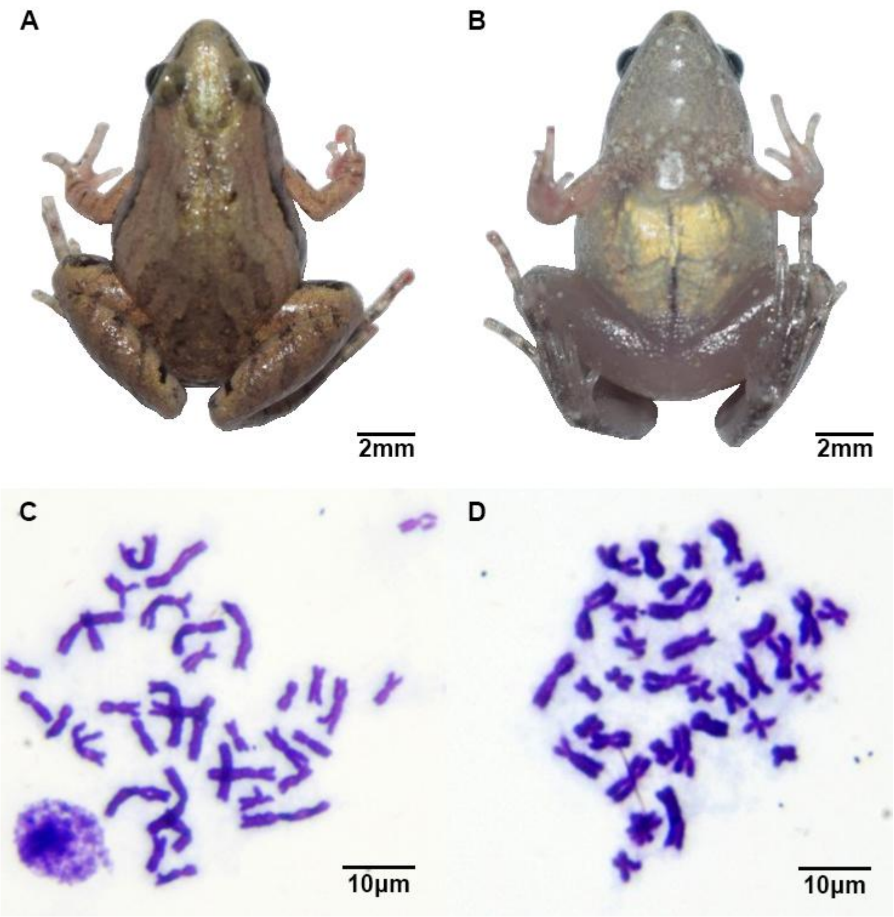

Figure S5 Triploid F1 generation (tetraploidy female × diploid male) obtained through hybridization. (A, B) Dorsal and ventral views of triploid individual at the age of five months, respectively. (C, D) Mitotic metaphase cell of triploid F1 individual.

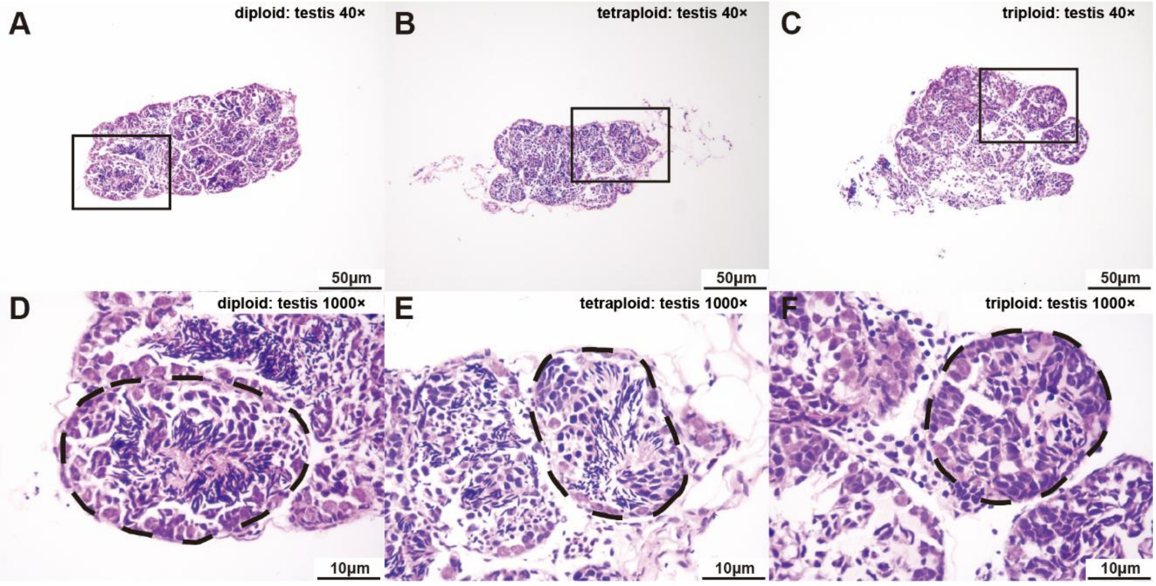

Figure S6 Comparison of the histological characteristics of testicle in diploid, tetraploid,

and triploid individuals (field of view under a low-magnification microscope). The seminiferous tubules are represented by dashed line region.

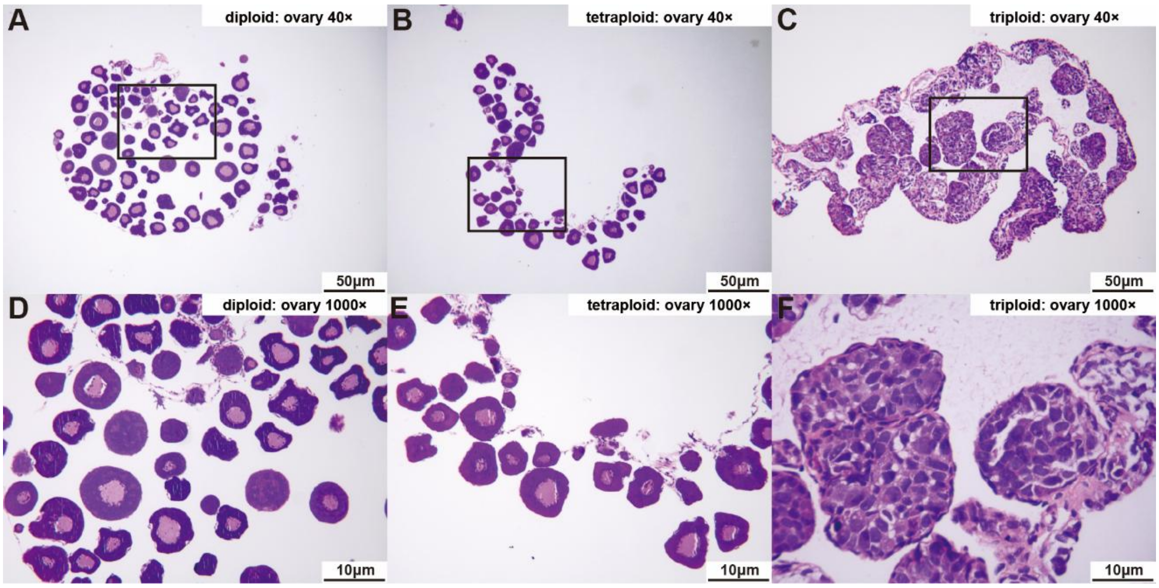

Figure S7 Comparison of the histological characteristics of ovary in diploid, tetraploid, and triploid individuals (field of view under a low-magnification microscope).

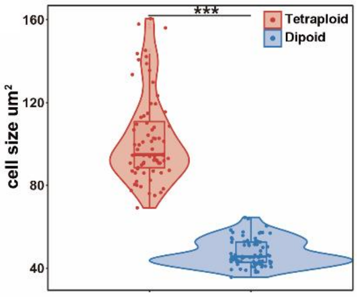

Figure S8 Comparison of the blood cell size. The data were analyzed using Mann-Whitney U test: ***, $p < 0.001$.

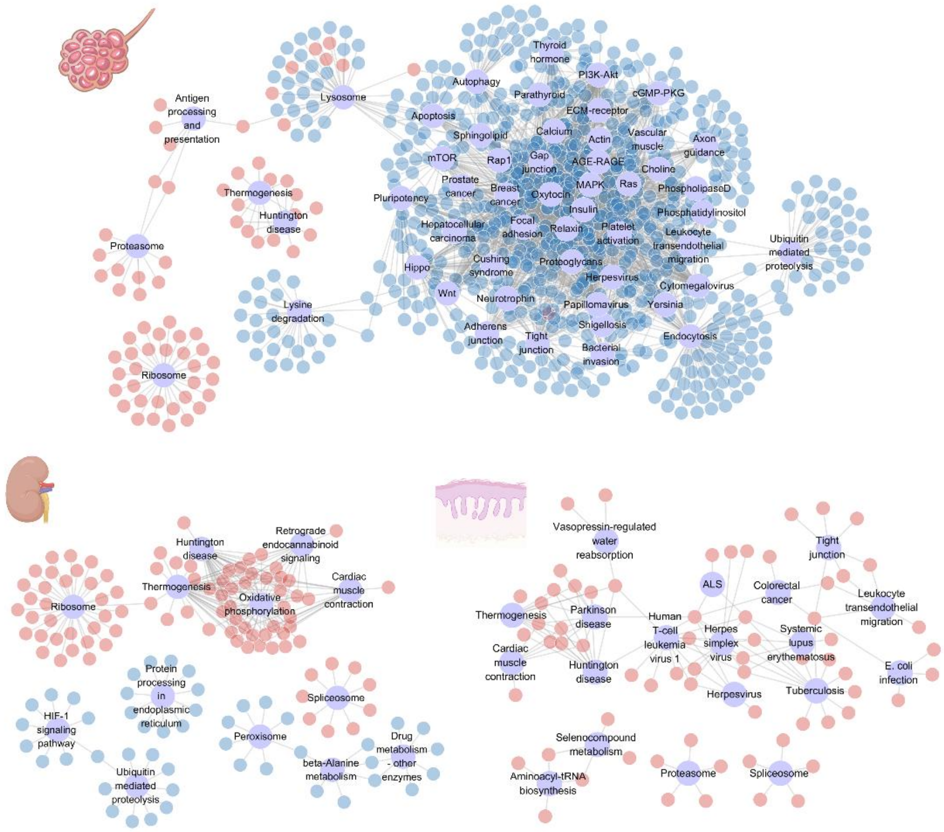

Figure S9 Networks illustrating the significantly differential KEGG pathways (adjusted p < 0.001) for each organ between diploid and tetraploid individuals. Large nodes represent KEGG items, while small nodes represent DEGs. Red and blue DEGs indicate higher transcriptional levels in tetraploid and diploid individuals, respectively. DEGs used for enrichment analyses had an adjusted p < 0.05 (Student's t-test and BH correction). For enrichment analyses based on downregulated DEGs in the lung, only the top 50 KEGG items (more stringent than adjusted p < 0.001) were used to construct the network, highlighting the most significant pathways. For the brain, liver, heart, muscle, and gonads, no KEGG items met the threshold due to the small number of DEGs. Please note that thermogenesis, Parkinson's, and Huntington's diseases were primarily enriched by genes involved in oxidative phosphorylation, which might have been omitted during the simplification of this network (see details in the methods section).
